# Development of a Cartilage Oligomeric Matrix Protein Neo-Epitope Assay for the Detection of Intra-Thecal Tendon Disease

**DOI:** 10.3390/ijms21062155

**Published:** 2020-03-20

**Authors:** Roger Smith, Patrik Önnerfjord, Kristin Holmgren, Shacko di Grado, Jayesh Dudhia

**Affiliations:** 1 Dept of Clinical Sciences and Services, The Royal Veterinary College, Hawkshead Lane, North Mymms, Hatfield, Herts AL9 7TA, UK; shacko.d@gmail.com (S.d.G.); jdudhia@rvc.ac.uk (J.D.); 2 Department of Clinical Sciences, Rheumatology and Molecular Skeletal Biology, University of Lund, 22184 Lund, Sweden; patrik.onnerfjord@med.lu.se (P.Ö.); Kristin.Holmgren@med.lu.se (K.H.); 3 Anicura Regiondjursjukhuset Bagarmossen, Ljusnevägen 17, 128 48 Bagarmossen, Sweden

**Keywords:** cartilage oligomeric matrix protein (COMP), tendon, neoepitope, tendinopathy

## Abstract

The diagnosis of tendon injury relies on clinical signs and diagnostic imaging but imaging is subjective and does not always correlate with clinical signs. A molecular marker would potentially offer a sensitive and specific diagnostic tool that could also provide objective assessment of healing for the comparison of different treatments. Cartilage Oligomeric Matrix Protein (COMP) has been used as a molecular marker for osteoarthritis in humans and horses but assays for the protein in tendon sheath synovial fluids have shown overlap between horses affected by tendinopathy and controls. We hypothesized that quantifying a COMP neoepitope would be more discriminatory of injury. COMP fragments were purified from synovial fluids of horses with intra-thecal tendon injuries and media from equine tendon explants, and mass spectrometry of a consistent and abundant fragment revealed a ~100 kDa COMP fragment with a new N-terminus at the 78th amino-acid (NH_2_-TPRVSVRP) located just outside the junctional region of the protein. A competitive inhibition ELISA based on a polyclonal antibody raised to this sequence yielded more than a 10-fold rise in the mean neoepitope levels for tendinopathy cases compared to controls (5.3 ± 1.3 µg/mL (*n* = 7) versus 58.8 ± 64.3 µg/mL (*n* = 13); *p* = 0.002). However, there was some cross-reactivity of the neoepitope polyclonal antiserum with intact COMP, which could be blocked by a peptide spanning the neoepitope. The modified assay demonstrated a lower concentration but a significant > 500-fold average rise with tendon injury (2.5 ± 2.2 ng/mL (*n* = 6) versus 1029.8 ± 2188.8 ng/ml (*n* = 14); *p* = 0.013). This neo-epitope assay therefore offers a potentially useful marker for clinical use.

## 1. Introduction

Overstrain injury to tendons are a common occurrence in athletes [1,2,3,4] and can affect different tendons in different locations in humans, dogs, and horses. In the horse, the most common injuries affect the weight-bearing tendons of the front limb, such as the superficial digital flexor tendon, and are usually mid-substance, extra-thecal (outside a tendon sheath or bursa) and contained within an intact paratenon, with strong similarities to Achilles tendinopathy in humans [5]. In contrast, overstrain injuries to tendons within a synovial environment (intra-thecal, such as a tendon sheath or bursa), more commonly occur as defects affecting the border of the tendon and are therefore in communication with the synovial cavity. These intra-thecal digital flexor tendon injuries in the forelimb of the horse represents a good large animal model for rotator cuff tears of the shoulder in humans [6]. These open lesions results in the release of extracellular matrix proteins into the synovial cavity which can provoke inflammation [7] and, in addition, there is a poorly identified adverse effect of synovial fluid on the tendon internal matrix which results in the loss of cells adjacent to the tear and a failure to heal [8]. Even with the current treatment of choice, tenoscopic debridement, there is a high failure rate for the successful management of these intrathecal tendon injuries in both horses and humans [9,10,11].

Diagnostic ultrasonography has been the lynchpin for the diagnosis of extra-thecal tendon injuries in the horse where it is not only used to confirm the presence of an injury in a specific tendon but also to determine its severity, as this has been linked to prognosis. However, there are limitations to this imaging modality—it is impractical as a screening tool and epidemiological studies have confirmed that this imaging technique cannot be used for predicting injury [1]. Furthermore, ultrasonography has poor sensitivity for the diagnosis of tendon tears within the digital sheath [10], and other imaging modalities (such as MRI) have similar limitations for identifying mild tendon border defects in both horses and humans, so alternative methods of diagnosis are needed. In these situations, molecular markers detected in the blood or tendon sheath synovial fluid could provide additional objective information on tissue metabolism (e.g., effect of training), diagnosis of injury (e.g., severity and stage), prognosis, and appropriate choice and efficacy of treatment. In horses, the site of pain is routinely determined by injecting local anesthetic into the tendon sheath or bursa when synovial fluid can easily be concurrently sampled and so this is an ideal large animal model to evaluate synovial fluid markers in naturally-occurring injury.

When a tendon is damaged, there is both physical and enzymatic destruction of the extracellular matrix which releases proteins and protein fragments either into adjacent synovial fluid when there is a surrounding tendon sheath and/or into the blood for extra-thecal injuries. These released molecules gain access to the systemic blood supply either via direct release into the blood or, more frequently via the lymphatic drainage from the extracellular space. Within the lymphatics, protein fragments are filtered from the lymph at lymph nodes, usually allowing relatively little to enter the systemic blood supply which can then be sampled for analysis. The development of a useful clinical assay for a protein biomarker for tendon injury relies on identifying a protein that is released into either the tendon sheath synovial fluid (for intra-thecal injuries) or minimally filtered by the lymphatics so that detectable amounts are released into the blood. For a blood biomarker, the ideal protein candidate should be one specific for tendon tissue or with at least a restricted distribution, so that its presence in the blood would not be derived from other tissues. However, when sampled from synovial fluid around a tendon (from a tendon sheath or bursa), there are limited tissues from which the protein can be derived, making this restricted distribution less of a requirement. Further selectivity for indicating injury can be achieved if an assay is developed against cleavage fragments of proteins which are specific for tendon injury.

One candidate for such a molecular marker is Cartilage Oligomeric Matrix Protein (COMP), which satisfies many of the above criteria. COMP is a non-collagenous extracellular matrix protein found predominantly in soft tissues whose function is mainly to resist load—cartilage, tendon, ligament, intervertebral disc, and meniscus. Studies on osteoarthritis in both humans [12,13,14,15,16,17,18] and horses [19,20,21,22,23,24,25,26,27,28] indicate that COMP may be a good marker for certain joint diseases, while the analysis of fragmentation patterns in joint synovial fluid suggested that COMP fragments were the most sensitive indicators of disease [17,18,19,25,28]. Within tendon, COMP is especially enriched in mid-metacarpal region of superficial digital flexor tendon in young adult horse [29], which is the most common site of injury. The protein is only loosely bound within the tendon extracellular matrix and is readily lost from the tendon by exercise, and therefore likely to be released early in the disease process [30,31]. However, previous studies have indicated that quantification of the total amounts of COMP in the blood did not reflect the presence of extra-thecal tendon disease, most likely because of high normal levels in blood (~1 µg/mL) and the relatively low amounts of COMP present in this part of the tendon in more mature horses [32]. In contrast, it has been shown that COMP levels increase significantly in tendon sheath synovial fluid with intra-thecal injury [33], although with overlap between cases and controls except in the oldest horses. Therefore, in order to discriminate between COMP released during normal metabolism and that produced by injury, we hypothesized that injury would generate a unique cleaved fragment and that an antibody could be generated that only recognized this new neo-epitope on the cleaved COMP molecule, thereby providing a means to develop a highly sensitive and specific assay for tendon damage. We therefore analyzed the COMP fragment patterns from both synovial fluids recovered from horses which had an intra-thecal tendon injury confirmed by tenoscopy and from tendon explants after in vitro stimulation with load and the C-terminal heparin-binding fragment of fibronectin [34]. This allowed the identification of protein fragments that were released from the tendon after injury, which would represent the best candidates for a molecular marker.

## 2. Results

### 2.1. Identification and Analysis of Cartilage Oligomeric Matrix Protein (COMP) Fragments

**Figure 1:**
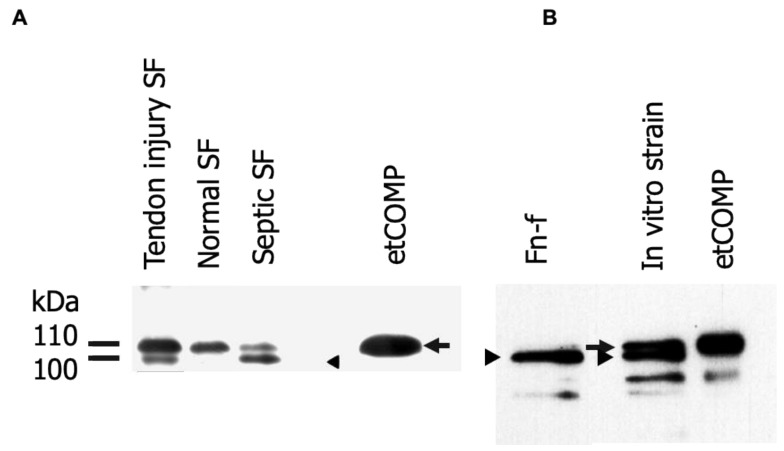
Western blots of 4–16% SDS-PAGE gels run under reducing conditions loaded with (**A**) semi-purified fragments from synovial fluid (SF) and (**B**) culture media from in vitro tendon explants subjected to treatment with Hep II (C-terminal heparin-binding) fragment of fibronectin (FN-f) or cyclical mechanical strain. The blot was labelled with a polyclonal anti-equine cartilage oligomeric matrix protein (COMP) antibody which recognizes intact as well as fragmented COMP. etCOMP = cartilage oligomeric matrix protein purified from equine tendon. Note the presence of a smaller fragment (arrow head) than the intact COMP monomer (arrow) only in abnormal synovial fluids and in media from tendon explants stimulated in vitro which was subsequently analyzed by MALDI-TOF mass spectrometry.

**Figure 2:**
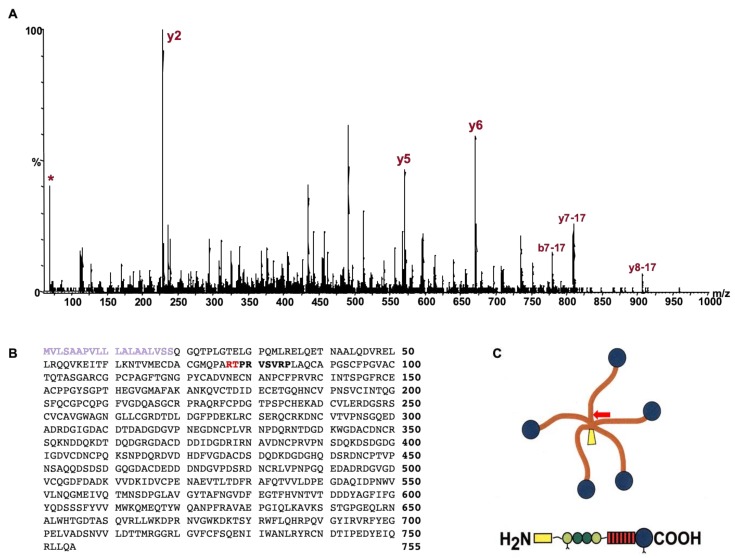
(**A**). Tandem mass spectrum of the neoepitope peptide “TPRVSVRPL” generated after chymotrypsin digestion and identified by mass spectrometry. The matching b- and y –ion fragments are labelled and the immonioum ion of proline is labeled with (*). (**B**) COMP sequence showing the cleavage site (in red) identified by mass spectrometry. The signal sequence is shown in purple. (**C**) Approximate location of the cleavage site after the 58th amino-acid and close to the disulphide bonds of the junctional region of COMP (arrow).

A number of fragments were identified in the semi-purified COMP fragments recovered from synovial fluid and media (Figure 1). The most prominent and consistent was a ~100 kDa fragment, just smaller than the intact COMP monomer, which was used for subsequent analysis with mass spectrometry. The most common enzyme used for proteomics is trypsin and the sequence coverage for the generated peptides indicated that the N-terminal part of COMP was missing in comparison to gel bands from intact COMP used as control. The most N-terminal peptide identified was “VSVRPLAQCAPGSCFPGVACTQTASGARCG” which is an expected tryptic peptide (data not shown). However, no peptide was found N-terminal to this site; neither with trypsin nor with Lys-C or Asp-N endoproteinases. Instead, we finally digested the COMP fragment using chymotrypsin which allowed a semi-specific cleaved (non-expected cleavage at one end) peptide to be identified. The MSMS spectrum (Q-TOF data) from this peptide is shown in Figure 2A which allowed the peptide sequence to be identified to “TPRVSVRPL” which also explains why trypsin digestion failed as the TPR peptide would not be long enough for detection. The COMP sequence is shown in Figure 2B highlighting the cleavage site as well as the neoepitope, while the overall COMP structure is depicted in Figure 2C.

### 2.2. Development of the COMP Neoepitope Assay

**Figure 3:**
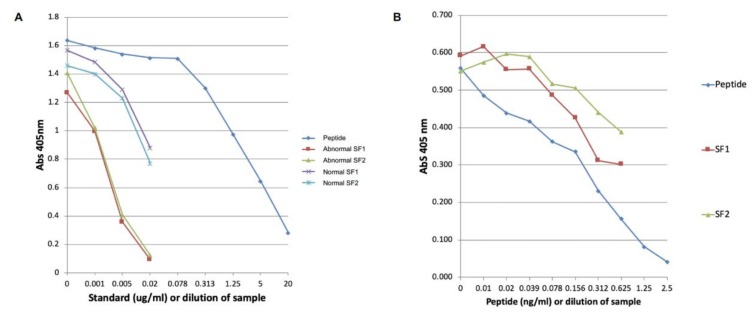
Parallel inhibition curves for (**A**) original neoepitope assay and (**B**) modified assay including spanning peptide to block cross-reactivity of the neoepitope antiserum with intact COMP. Both normal and abnormal synovial fluids show good parallel inhibition in both assays.

Dilutions of normal and abnormal digital sheath synovial fluids of 1/4 to 1/256 showed parallel inhibition curves with the peptide standard (Figure 3) indicating good specificity of the assay. Spiking synovial fluid with the peptide showed parallel inhibition with the peptide standard. However, if the synovial fluid was diluted by more than 1 in 20, then parallel inhibition was not observed (data not shown).

**Figure 4:**
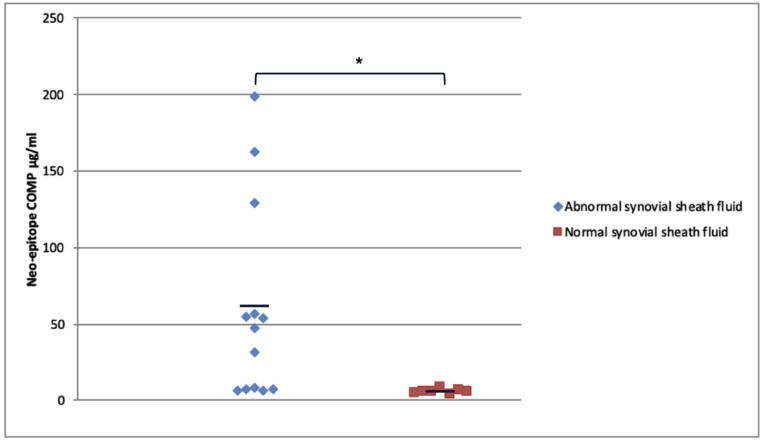
Quantification of the COMP neoepitope in both normal (*n* = 7) and abnormal (from cases with intra-thecal tendon injury; *n* = 13) tendon sheath synovial fluids. The bars show the mean value. * denotes significant difference between the groups; *p* < 0.002. Note the large variation seen for the abnormal synovial fluids.

Normal digital sheath synovial fluid (*n* = 7) contained on average 5.3 ± 1.3 µg/ml of COMP neo-epitope. In contrast horses with intra-synovial tendon injuries (*n* = 13) had COMP neo-epitope levels of 58.8 ± 64.3 µg/mL (Figure 4) which was significantly different from the controls (*p* = 0.002). However, there was large variation between the abnormal samples, ranging from 5.6–198.6 µg/mL.

### 2.3. Cross-Reactivity of the Neo-epitope Antibody with Intact COMP

**Figure 5:**
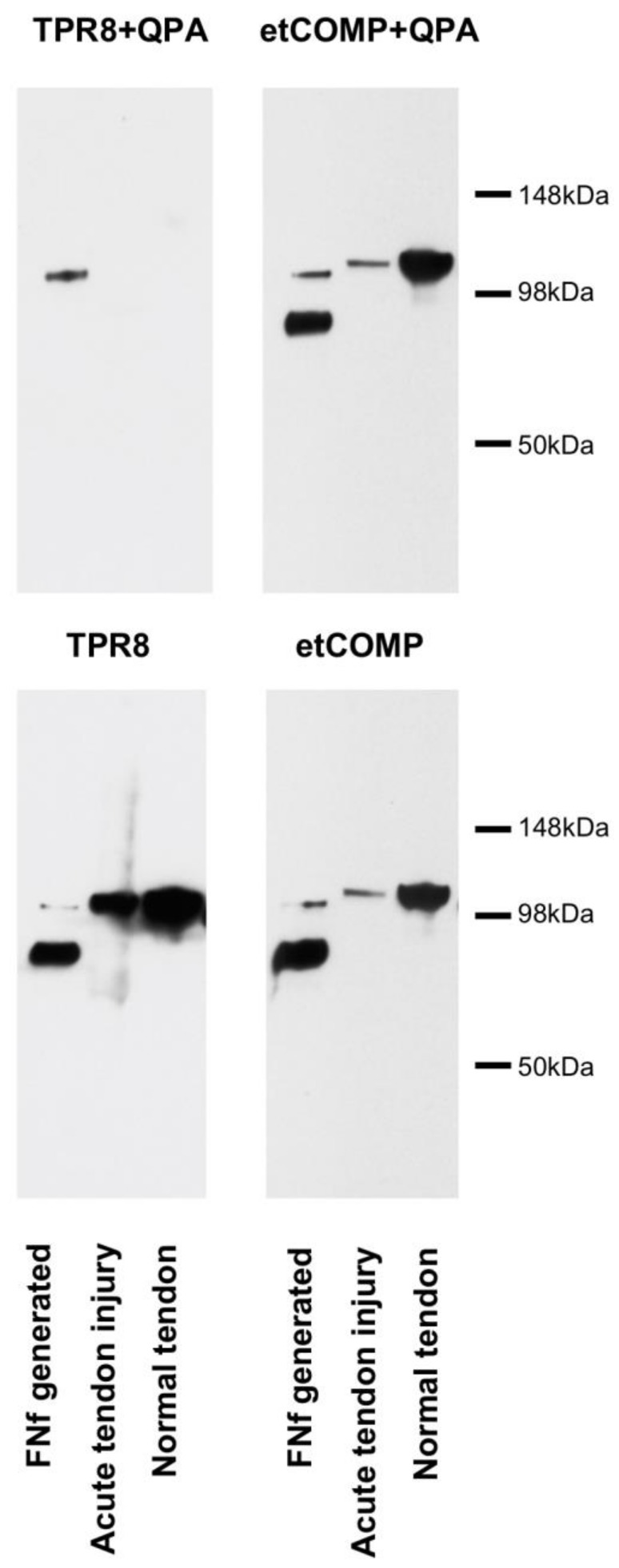
Identical western blots of 4–16% SDS-PAGE gels run under reducing conditions, probed with a polyclonal antibody raised against equine COMP purified from equine tendon (etCOMP) neoepitope polyclonal antiserum (TPR8), with (top row) and without (bottom row) the spanning peptide (QPA) to reduce cross-reactivity of the neoepitope anti-serum to non-cleaved COMP molecule. Note the addition of the spanning peptide has minimal effect on the labelling with the polyclonal antiserum to the whole molecule, while it blocks binding of the neoepitope antiserum to all other COMP bands apart from the ~100 kDa fragment from which the neoepitope antiserum was developed.

Western blots probed with the neo-epitope polyclonal antiserum and a polyclonal antiserum against whole COMP showed that the neo-epitope antiserum cross-reacted with intact COMP. However, this reactivity could be blocked by including a 13 amino-acid spanning peptide (Figure 5).

### 2.4. Modified COMP Neo-Epitope Assay

**Figure 6:**
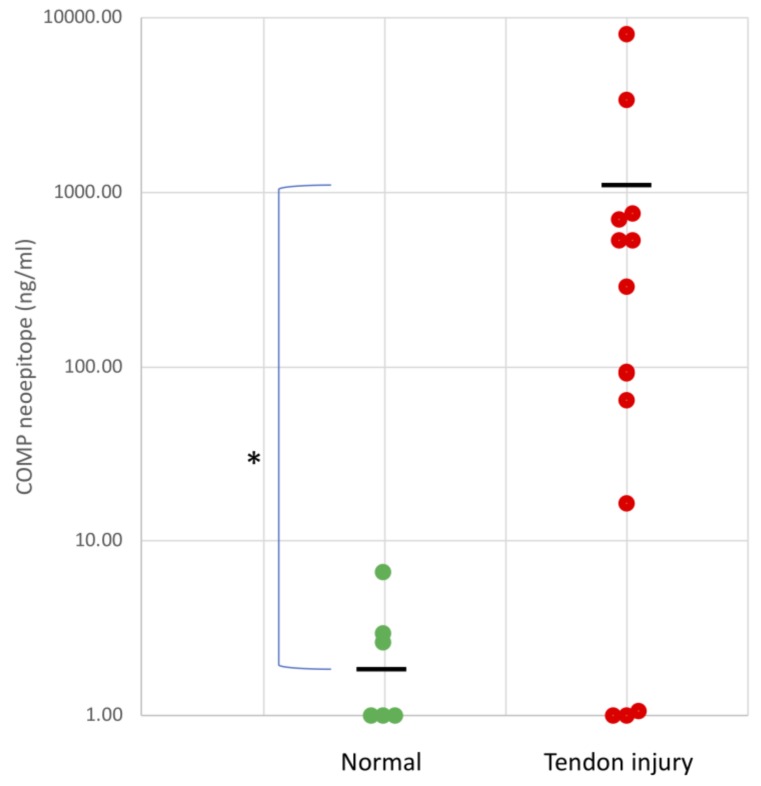
Quantification of the neoepitope in synovial fluids from normal tendon sheaths (*n* = 6) and from tendon sheaths containing tendon injury (*n* = 14). Samples with values under the detectable range of the assay were given a value of 1 ng/mL which is the limit of detection of the assay. Lines show the mean levels for each group. Note the logarithmic scale on the y axis to enable comparison between normal and cases and the large difference in neoepitope concentration between normal (<10 ng/mL) and those with injury (an average of 500-fold higher; * *p* = 0.013).

Using the spanning peptide, normal digital sheath synovial fluid (*n* = 6) contained minimal quantities of neoepitope (2.5 ± 2.2 ng/mL) with three out of the six samples having values below the detectable range of the assay (1 ng/mL). In contrast, synovial fluids from horses with intrathecal tendon injuries (*n* = 14) had significantly higher values (1029.8 ± 2188.8 ng/mL; *p* = 0.013; Figure 6) but with a large variation.

### 2.5. Relationship of Neo-Epitope Concentration to Total COMP

**Figure 7:**
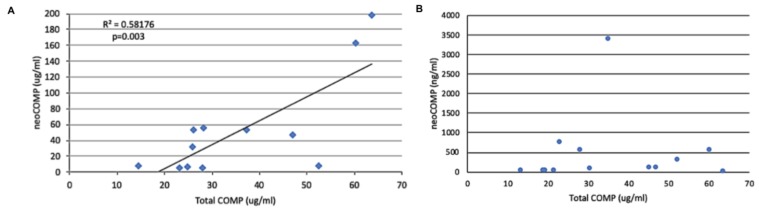
The relationship between the total COMP content of the tendon sheath synovial fluids (determined using a homologous inhibition assay) and concentration of the COMP neoepitope determined by the original assay (**A**) and the modified assay (**B**) containing the spanning peptide to block cross-reactivity of the neoepitope antiserum with intact COMP. Note the significant relationship for the original assay, reflecting the cross-reactivity of the neoepitope antiserum but the lack of relationship with the cross-reactivity blocked.

The values for total COMP were 38.6 ± 14.7 µg/mL for the cases with tendon injury compared to 17.2 ± 4.5 µg/mL for the controls. This was significantly different (*p* = 0.003). When these values were compared to the neo-epitope values for the original assay there was a significant relationship between the two (Figure 7). However, when the comparison was made with the modified assay using the spanning peptide to limit the cross-reactivity, there was no such correlation.

## 3. Discussion

This study has identified a COMP fragment that is released as a consequence of tendon injury. A number of fragments were identified, but the most prominent and consistent one was chosen to determine the cleavage site. Mass spectrometry has enabled the detection of multiple protein fragments from which neo-epitope assays can be developed. This technology was used to identify this site which is unique compared to the other cleavage sites that have been identified to date in the COMP molecule [17,35,36]. This is partly because the sequence in this region of the COMP molecule differs from other species but also because the site is adjacent to an arginine so that trypsin, commonly used for MALDI-TOF analysis, cleaves the fragment adjacent to the cleavage site so that there are no fragments with a new C terminus are identified. Chymotrypsin was the only enzyme that enabled the identification of the cleavage site. However, interestingly, a similarly sized fragment was identified in vitro in preparations of recombinant human COMP which was found to be a cleavage at amino-acid 78, the same as in this study [37], although the sequence in human COMP at this site is slightly different. This cleavage was speculated to be due to an unknown protease in the preparation. Candidate enzymes capable of performing the cleavage was investigated using on-line tools such as MEROPS and PeptideCutter, but was unsuccessful. Although not the same protein, MALDI-TOF mass spectrometry of the cleavage of collagen IX with MMP-13 has shown more than one fragment with a new C terminus, PAR [38], which is identical to the cleavage site identified in this study in COMP which raises the possibility that this is also an MMP-13 cleavage site. The fact that the protein is released early both in vivo and in vitro suggests that this release is a consequence of tissue damage rather than repair. 

The results obtained in this investigation support the hypothesis that an assay can be developed using a neo-epitope antiserum against a cleavage site in COMP, capable of distinguishing horses with intra-synovial tendon pathology from those with no injury. The results for the initial neoepitope assay showed that there is a statistically significant difference in neo-epitope COMP levels in horses with intra-synovial tendon injury compared to normal horses. The average increase was more than 10-fold which compares favorably with the 1.5-fold elevation of total COMP using the previously documented COMP ELISA assay [33]. However, the neo-epitope antiserum was shown to cross-react with the intact molecule and so the assay was modified by adding a spanning peptide to cover the cleavage site. This resulted in a marked reduction in the concentration of the neo-epitope but the average concentration for the synovial fluid samples from tendon injuries now showed more than a 500-fold increase compared to the controls. This is consistent with our previous observations that fragmented COMP is released into the digital sheath synovial fluid from the tendon post-injury and supports the hypothesis that measuring fragments offers a more discriminatory assay for tendon injury.

However, the results do show considerable variation between cases, with not all samples showing an increase and therefore resulting in overlap in COMP neo-epitope levels in digital sheath synovial fluid of horses with tendinopathies compared to horses with no injury. This might be explained by some samples of digital sheath synovial fluid used in this study being from horses with less severe tendon injuries or from a different types of injury which did not result in significant COMP fragmentation. The tendon pathologies identified involved both the superficial (tears of the manica flexoria) and deep (longitudinal tears) digital flexor tendons, but subanalysis of the different pathologies did not show any significant differences. It is not clear what pathologies induce the most fragmentation although proteomic analysis of COMP fragmentation patterns from equine tendon show injury-specific fragments which could also be induced by treatment of tendon explants in vitro with inflammatory cytokines [35]. Therefore, these markers may be more elevated when there are greater degrees of inflammation. This was supported by a previous investigation in COMP release into digital sheath synovial fluid where total COMP concentration was greatly increased by the presence of sepsis which is highly inflammatory [32]. However, fragmentation patterns may be different with sepsis as one case of tendon sheath sepsis was analyzed, the levels were below the detection limit (data not shown) and so an assay specific for a particular neo-epitope may not always been increased in all pathologies.

An additional explanation to why some COMP neo-epitope levels were not increased despite being sampled from horses with intra-synovial tendon injuries may be due to differing amounts of effusion or hemorrhage within the sheath which could dilute and lower the COMP fragment levels. By calculating the dilution of the marker from the ratio of extra fluid in the effusion to normal volume of digital sheath synovial fluid, or by expressing it against a marker that does not alter with disease [39], this problem could be eliminated. However, data for total COMP from a variety of joint diseases have shown minimal changes in COMP levels, even in the presence of significant effusion associated with disease [22,26,40], suggesting that the total COMP concentrations are independent of effusion and so can be used as a relatively constant denominator for a ratio calculation. However, when neoepitope values were expressed as a percentage of the total COMP, there was still a large variation which did not result in significant difference between the groups.

Additionally, the duration of injury may also influence COMP neo-epitope levels detected in digital sheath synovial fluid. COMP levels have been observed to fall in chronic osteoarthritis in both humans and horses [22,26] while they are elevated in acute joint damage [27]. This is thought to be because of depletion of the COMP from the cartilage in chronic disease. A similar situation may occur with tendon tears where COMP from within the tendon can leach out over time and deplete the levels in the tendon, especially in the compressed regions where the levels are highest in adult horses [29] and where the tears are most commonly found [10,41]. It was not possible to relate the levels to the duration of the tendon lesions in this study. Communication of the tendon lesion within the synovial cavity is likely to increase the release of fragments into the synovial fluid and certainly the highest levels of the neoepitope was seen in a border tear of the deep digital flexor tendon, while a contained lesion affecting the same tendon had levels below the detectable range. COMP levels are maintained in those areas within the digital flexor tendon sheath [29] and age of the patient has minimal effect on the total COMP levels in tendon sheath synovial fluid post skeletal maturity [33]. Therefore, because of this and the fragment identified for use in the neoepitope assay only being detected with injury, age is unlikely to have had a significant influence on COMP neo-epitope levels. 

These influences could explain the three case samples which had low levels of the neo-epitope. One case had a core lesion within the tendon with no communication to the tendon sheath which might explain the lack of release of the neoepitope into the synovial fluid. The second had only a partial tear of the manica flexoria which represented a mild injury, and the third was a tear of the deep digital flexor tendon but of unknown duration. 

This study’s objective was to develop a neo-epitope assay for a cleaved fragment of COMP that was generated through tendon damage and show that it could be used to detect this fragment in synovial fluids from tendon sheaths which contained naturally-occurring tendon injury. The wide variation seen between the samples suggests that the assay is best at detecting a subset of horses with tendon injuries which may be useful for diagnostic, therapeutic, or prognostic purposes. 

Based on this data, however, it is not possible to state if the fragment is unique to tendon and, indeed, additional preliminary data (unpublished) have suggested that it is also generated from cartilage, another tissue containing high levels of COMP, when analyzing synovial fluid from joints. The source of the synovial fluid therefore can be used to differentiate tendon from cartilage pathology (tendon sheaths and bursae do not contain cartilage) and so the marker may also be useful for diagnosing or monitoring joint disease. Therefore, the clinical signs would also be important if the marker was to be analyzed in blood to differentiate different sources of the fragment. The assay has been able to detect the fragment in a limited number of serum samples (data not shown). However, there are still many challenges regarding the understanding and measurement of molecular markers. Rates of formation, accumulation, and clearance of biomarkers from digital sheath synovial fluid and serum are still unclear and local factors, such as local inflammation, may have addition influence on the levels of markers. Further work will therefore be necessary to establish its clinical usefulness on a larger number of clinical cases.

## 4. Materials and Methods

### 4.1. Determination of COMP Cleavage Site

COMP fragments were partially purified from a number of sources using ion-exchange chromatography as described for tendon extracts [29]:

Normal digital sheath synovial fluids

Inflammatory synovial fluid from the digital flexor tendon sheath

Synovial fluid from infected digital flexor tendon sheath

Media from tendon explants undergoing cyclical mechanical loading and with and without stimulation by a C-terminal fragment of heparin [34]

Fragments that were consistently released from tendon in greatest amounts with injury were identified by Western blotting using anti-equine COMP polyclonal antisera [33] (Figure 1).

### 4.2. Identification of Cleavage Site Using Mass Spectrometry

MALDI-TOF mass spectrometry using trypsin, chymotrypsin, endoproteinase Lys-C, and Asp-N digestion of the most abundant cleaved COMP from SDS-PAGE separation of the cleaved proteins from tendon explants in culture was used to identify the cleavage site. These digest ‘maps’ were compared with the digestion of intact COMP to identify those with a new N-terminal sequence indicative of the cleavage site. Liquid chromatography mass spectrometry (LC-MS) using a quadruple time-of-flight mass spectrometer (Q-TOF micro, Waters) were performed on the chymotrypsin digest of the COMP fragment to identify the N-terminal neo-epitope peptide i.e. the cleavage site. 

### 4.3. Generation of Neo-Epitope Antiserum

An 8 amino-acid peptide (NH2-TPRVSVRC-COOH; abbreviated to TPR-8)) which matched the sequence of the N terminal side of the cleavage (representing the new N terminus of a COMP fragment released after injury) were synthesized, and bound to keyhole limpet haemocyanin KLH, then used to immunize a rabbit in conjunction with Freunds Complete Adjuvant. Booster immunizations were performed at weeks 3, 5, 8, and 11 with Freunds Incomplete Adjuvant. 

### 4.4. Quantification of NeoCOMP in Digital Sheath Synovial Fluid

Digital sheath synovial fluid samples were obtained from 20 horses with intra-thecal tendon disease, and 13 controls, as indicated in Table 1. All clinical cases underwent clinical examination, diagnostic analgesia, ultrasonographic examination, and tenoscopy to determine the exact diagnosis. Controls were from fresh cadavers which, after synovial fluid aspiration, were inspected by dissection. Seven normal samples were obtained from an abattoir from horses with no history or clinical signs of tendon or ligament injury which was confirmed postmortem by dissection. The synovial fluids were either sampled directly from clinical cases at the time of intra-thecal analgesia, or immediately post-mortem in normal horses (within 10 min). The synovial fluid samples were centrifuged to remove cells and were stored at −20 °C until used in the assay.

A 96-well Maxisorp™ ELISA plate (Nunc) was coated with 1 μg/mL of TPR-9 (9 amino-acid peptide; NH_2_-TPRVSVGGGC) in 50 mM sodium carbonate (pH 9.5) and incubated overnight in a dark humidified chamber. Simultaneously standards of freeze-dried TPR-9 (5 μg/mL to 0.005 μg/mL) were dissolved in 0.8% sodium dodecyl sulphate (SDS) in phosphate buffered saline (PBS) (pH 7.4), with 0.5% bovine serum albumen (BSA). Using a Sterilin™ 96-well plate, 50 μL of each standard dilution (in duplicate) was added to each well. Development of the assay included quality control steps of using serial dilutions of the peptide from 1/1280–1/10 and parallel inhibition curves for the synovial fluids. Dilutions of the normal and abnormal synovial sheath fluids (not treated to reduce viscosity) were prepared in the same sample buffer. 

After the overnight incubation at room temperature, the coated Nunc wells were washed four times with wash buffer (150 mM sodium chloride, 0.05% Tween-20) and blocked with 2 mg/mL BSA in PBS (pH 7.4). At the same time, 50 μL of antibody TPR-8 (concentration 1:800 in 4% Triton X-100 in 10 mM Sodium Phosphate, pH 7,4) without or with 20 ug/mL of a 13 amino-acid peptide which spans the cleavage site (QPARTPRVSVRPL; blocking peptide used to limit the cross-reactivity of the neo-epitope antiserum with the intact protein in the modified assay), was added to the Sterilin™ plate. Both plates were then incubated for 1 h in a dark humidified chamber. After 1 h the Nunc plate was washed four times with wash buffer and 100 μL of the contents of the Sterilin™ plate was transferred to the respective wells on the coated plate. The plate was incubated for 1 h in a dark humidified chamber at room temperature. Next the plate was washed four times with wash buffer and 50 μL of secondary antibody (alkaline phosphatase-conjugated swine anti-rabbit IgG; Dako; diluted 1:1000 in 0.14 M NaCl, 0.008M Na_2_HPO_4_, 2.8 mM KCl, 1.4 mM KH_2_PO_4_, 3 mM NaN_3_, pH7.4 with 0.01% Tween-20 and 2 mg/mL BSA was added to each well. The plate was incubated for a further hour. Subsequently, the plate was washed four times with wash buffer and then 100 μL of 1 mg/mL paranitrophenylphosphate in substrate buffer (1 M diethanolamine buffer in 0.5 mM MgCl_2_, pH 9.8) was added to each well. The first reading was taken at 405 nm immediately using an Expert 96 (Asys Hitech) ELISA plate reader (time 0). The plate was then kept in the dark and gently agitated for 60 minutes before the second reading was obtained (time 1 h) to provide a 2-point kinetic measurement. MikroWin version 4.23 software (Siemens) was used to draw the standard curve and used for quantification of COMP neo-epitope. 

### 4.5. Quantification of Total COMP in the Same Tendon Sheath Synovial Fluids

This was quantified using a homologous inhibition assay developed in house and previously described (32). This used COMP purified from equine tendon as the plate coat and the standards and a polyclonal antiserum against equine tendon COMP as the primary antibody. The solutions and protocol were identical to that described above for the original neoepitope assay.

### 4.6. Statistical Analysis

The normal and abnormal digital sheath synovial fluid samples were compared to establish whether there was a significant difference in the mean COMP neo-epitope levels between horses with intra-synovial tendon injuries and control horses. Data was tested for normality (or normal distribution) and significance between groups was tested using the Mann-Whitney U test (SPSS version 26 software) and differences considered significance for *p* < = 0.05.

## 5. Conclusions

This study has identified a cleaved fragment of COMP from which a neo-epitope inhibition ELISA has been successfully developed. We have shown that this assay is able to detect the cleavage fragment in tendon sheath synovial fluids of some, but not all, cases of naturally-occurring intra-thecal tendon injury, showing marked rises compared to normal synovial fluids. This neo-epitope therefore appears to be a more suitable for clinical use than previous ones evaluated for tendon disease [32,33,42]. The development of a serological assay for tendon injuries could allow an objective method of diagnosing tendon injuries in horses (and humans) and provide an opportunity to detect and therefore investigate the early disease process of tendon pathologies as well as improving monitoring, prognosis and objectively evaluating the efficacy of different treatments. The assay, however, shows a high variability and further studies are needed to address the current data’s limitations and to prove its usefulness as a diagnostic tool.

## Figures and Tables

**Table 1:** Details of the 33 digital flexor tendon sheath (DFTS) synovial fluid samples analyzed with the three different assays.

Tendon Injury	Age (Years)	DFTS Pathology	Total COMP Assay	Neoepitope Assay	Modified Neoepitope Assay
1	11	MF	x		x
2	9	DDFT	x		x
3	13	DDFT	x		x
4	7	DDFT	x		x
5	19	MF	x	x	
6	7	DDFT	x	x	x
7	14	DDFT	x	x	x
8	10	MF	x	x	x
9	14	MF	x	x	
10	12	MF	x	x	
11	4	DDFT	x	x	
12	ND	MF	x	x	x
13	11	DDFT	x	x	
14	15	MF	x		x
15	ND	MF	x	x	x
16	5	DDFT (core)	x	x	x
17	16	SDFT	x	x	x
18	12	MF	x	x	
19	ND	DDFT	x		x
20	ND	DDFT	x		x
n			20	13	14
**Controls**					
1	Aged	Normal	x	x	
2	Middle	Normal	x	x	
3	Middle	Normal	x		x
4	Middle	Normal	x		x
5	Middle	Normal	x		x
6	Middle	Normal	x		x
7	Aged	Normal	x		x
8	Aged	Normal	x		x
9	1	Normal	x	x	
10	3	Normal	x	x	
11	Middle	Normal	x	x	
12	4	Normal	x	x	
13	ND	Normal	x	x	
n			13	7	6
					
**Total Number of Samples**			**33**	**20**	**20**

Age—Control samples were recovered from normal fresh cadaveric limbs and ageing of the horse was usually only possible by dentition which is relatively inaccurate. Hence, ages are given as ‘Middle’ for horses approximately 5–12 years of age and ‘Aged’ for horses approximately 13–25 years. If documentation of the horse’s age was available it was recorded; ND = not determined; Diagnosis—MF = longitudinal tear of the superficial digital flexor tendon’s manica flexoria; DDFT = longitudinal tear of the deep digital flexor tendon; DDFT (core)—central longitudinal disruption of the tendon but with no communication to the outside of the tendon (i.e., surrounded by intact tendon tissue); SDFT = longitudinal tear of the superficial digital flexor tendon.
